# Development and validation of a predictive model for invasive ventilation risk within 48 hours of admission in patients with early sepsis-associated acute kidney injury

**DOI:** 10.3389/fmed.2025.1577154

**Published:** 2025-06-18

**Authors:** Li Hong, Bin Wang

**Affiliations:** ^1^Department of Infectious Diseases, Affiliated Dongyang Hospital of Wenzhou Medical University, Dongyang, Zhejiang, China; ^2^Department of Emergency, Affiliated Dongyang Hospital of Wenzhou Medical University, Dongyang, Zhejiang, China

**Keywords:** sepsis, acute kidney injury, invasive ventilation, prediction model, machine learning

## Abstract

**Objective:**

To identify patients with early sepsis-associated acute kidney injury (SA-AKI) at high risk of requiring invasive ventilation within 48 h of admission, facilitating timely interventions to improve prognosis.

**Methods:**

This retrospective study included patients with early SA-AKI admitted to Dongyang People’s Hospital between January 2011 and October 2024 and Yiwu Tianxiang Dongfang Hospital between January 2016 and December 2024. Variables included age, blood parameters, and vital signs at admission. Patients were divided into training and validation cohorts. Independent risk factors were identified in the training cohort, and a nomogram was developed. The discriminatory ability was assessed using the area under the receiver operating characteristic curves (AUC). Calibration was assessed using GiViTI calibration plots, while clinical utility was evaluated via decision curve analysis (DCA). Validation was performed in the internal and external validation groups. Additional models based on Sequential Organ Failure Assessment (SOFA) and National Early Warning Score (NEWS) scores, machine learning models including Support Vector Machine (SVM), C5.0, Extreme Gradient Boosting (XGBoost), and an ensemble model were compared with the nomogram on the discrimination power using DeLong’s test.

**Results:**

The key independent risk factors for invasive ventilation in patients with early SA-AKI included lactate, pro-BNP, albumin, peripheral oxygen saturation, and pulmonary infection. The nomogram demonstrated an AUC of 0.857 in the training cohort (Hosmer-Lemeshow *P* = 0.533), 0.850 in the inner-validation cohort (Hosmer-Lemeshow *P* = 0.826) and 0.791 in the external validation cohort (Hosmer-Lemeshow *P* = 0.901). DCA curves indicated robust clinical utility. The SOFA score model exhibited weaker discrimination powers (training AUC: 0.621; validation AUC: 0.676; *P* < 0.05), as did the NEWS score model (training AUC: 0.676; validation AUC: 0.614; *P* < 0.05). Machine learning models (SVM, C5.0, XGBoost, and ensemble methods) did not significantly outperform the nomogram in the validation cohort (*P* > 0.05), with respective AUCs of 0.741, 0.792, 0.842, and 0.820.

**Conclusion:**

The nomogram developed in this study is capable of accurately predicting the risk of invasive ventilation in SA-AKI patients within 48 h of admission, offering a valuable tool for early clinical decision-making.

## Introduction

Sepsis is a life-threatening condition caused by an uncontrolled host response to infection, leading to organ dysfunction (1, 2). Despite advances in management, its incidence and mortality remain high (3). Indeed, in recent decades, the prevalence of sepsis has been rising (4), and it now poses a significant threat to global public health (5–7).

Acute kidney injury (AKI) frequently develops in sepsis patients, particularly within the first 48 h of admission, a condition termed early sepsis-associated acute kidney injury (SA-AKI) (8, 9). These patients face a heightened risk of respiratory failure, often necessitating invasive ventilation, which significantly increases mortality and prolongs hospitalization (10). Although the implementation of the Sepsis Rescue Campaign has led to improvements in sepsis diagnosis and treatment, a subset of patients still require mechanical ventilation (11, 12). Studies have shown that early mechanical ventilation in sepsis patients is linked to poor prognosis (2), highlighting the need for an effective prediction model to identify SA-AKI patients at high risk of early mechanical ventilation, thereby potentially enabling timely interventions to improve outcomes.

Traditional tools used to predict sepsis severity and disease progression include the Sequential Organ Failure Assessment (SOFA) score (13), National Early Warning Score (NEWS) (14), and Acute Physiology and Chronic Health Evaluation (APACHE) II score (15). While they can effectively assess the risk of organ failure in sepsis patients, they were not designed to predict invasive ventilation risk in SA-AKI patients. Existing models have been developed to predict acute respiratory distress syndrome (ARDS) risk in sepsis patients (16, 17), yet they do not encompass all cases requiring invasive ventilation. Other models predict respiratory failure and mechanical ventilation risk in sepsis patients (18, 19), but their applicability to the SA-AKI subgroup remains unclear.

To date, there is no dedicated model for predicting short-term invasive ventilation risk in SA-AKI patients. Therefore, this study aimed to develop a model for predicting mechanical ventilation risk within 48 h of admission in SA-AKI patients. The implementation of such a model may provide support for clinical decision-making and improve doctor-patient communication.

## Materials and methods

### Study population

The retrospective study population included sepsis patients admitted to Dongyang People’s Hospital from January 2011 to October 2024 as training and internal validation groups and cases from Yiwu Tianxiang Dongfang Hospital from January 2016 to December 2024 as the external validation group. Inclusion criteria: (1) Diagnosis of sepsis according to Sepsis-3.0 criteria (defined as the presence of infection with a SOFA score increase of ≥ 2 points); and (2) meeting the criteria for AKI (defined by serum creatinine > 26.5 mmol/L, a ≥ 50% increase from baseline within 48 h of admission, or urine output < 0.5 mL/kg/h for more than 6 h). Exclusion criteria: (1) Patients < 18 years of age; (2) patients with uncertain baseline renal function (3) patients having withdrawn from treatment; and (4) patients that underwent emergency abdominal surgery.

### Data collection

Patient demographics, including sex, age, and medical history, were recorded. Comorbidities assessed included diabetes, cerebral infarction, chronic lung diseases (such as COPD, interstitial pneumonia, and pulmonary fibrosis), chronic liver disease (cirrhosis), hypertension, chronic heart disease (NYHA class II or higher), chronic kidney disease (chronic renal insufficiency and nephrotic syndrome), leukemia, and malignancy. Laboratory data and vital signs at admission were collected, including high-sensitivity C-reactive protein, total bilirubin, aspartate aminotransferase, triglycerides, creatinine, lactate, cholinesterase, pro-BNP, D-dimer, prothrombin time, sodium, potassium, magnesium, calcium, white blood cell count, hemoglobin, platelets, albumin, and globulin. Vital signs recorded included peripheral oxygen saturation, body temperature, heart rate, mean arterial pressure, respiratory rate, and Glasgow Coma Scale (GCS) score. Infection sites were categorized as intracranial, gastrointestinal, biliary, pulmonary, or urinary tract infections. Criteria for invasive ventilation included: (1) persistent dyspnea, tachypnea (> 30 breaths/min), PaO_2_ < 50 mmHg, or PaO_2_/FiO_2_ < 200 despite aggressive treatment (including medicated oxygen therapy, high-flow oxygen therapy, or non-invasive ventilation); (2) respiratory depression (< 8 breaths/min); (3) impaired consciousness, including stupor or coma; (4) progressive hypercapnia with pH ≤ 7.20; (5) persistent circulatory failure despite treatment.

### Variable selection

The patients with missing data across all the enrolled variables were less than 25% and interpolated by multiple imputation using “mice” packages. Then, continuous variables were transformed into categorized ones based on normal ranges. The dataset from Dongyang People’s Hospital (center 1) was randomly divided into a training group (70%) and a internal validation group (30%), and the patients from Yiwu Tianxiang Dongfang Hospital (center 2) was designed as the external validation group. Univariate analysis (*P* < 0.01) identified significant variables in the training group. Multicollinearity was assessed using variance inflation factors (VIF < 10). The Box-Tidwell test was performed to confirm no linear relationship between variables and logit(p) (*P* < 0.05). Independent risk factors were identified using multivariate logistic regression and stepwise regression and subsequently incorporated into the nomogram (Supplementary Figure 1).

### Model development, validation, and evaluation

The model’s discriminative ability was evaluated using Receiver Operating Characteristic (ROC) curve analysis, with the Area Under the Curve (AUC) serving as the primary metric (AUC > 0.75 indicating strong discrimination). Optimal cutoff values were determined using Youden’s index, and sensitivity, specificity, Positive Predictive Value (PPV), and Negative Predictive Value (NPV) were calculated. Calibration was assessed using the Hosmer-Lemeshow test (*P* > 0.05 indicating good calibration). Clinical effectiveness was evaluated using Decision Curve Analysis (DCA), with effectiveness determined by the model curve being distinct from the extreme reference curves.

Sequential Organ Failure Assessment and NEWS-based predictive models were developed and compared to the nomogram using Delong’s test (*P* < 0.05 indicating statistical significance). Machine learning models [Support Vector Machine (SVM), C5.0, Extreme Gradient Boosting (XGBoost), and Ensemble] were constructed in the validation group. An integrated model was developed using the Stacking method and compared to the nomogram using Delong’s test.

### Statistical analysis

The required minimum of sample size was evaluated based on the numbers of enrolled variables and patients requiring ventilation using “pmsampsize” package in R software (version 4.1.2). Normally distributed continuous variables were expressed as mean ± standard deviation (SD) and compared using *t*-tests. Non-normally distributed variables were reported as means and interquartile ranges (IQR), and analyzed using the Mann-Whitney U test. Categorical variables were expressed as percentages and compared using chi-square tests. All statistical analyses were performed using R software.

## Results

### Baseline patient characteristics

A total of 769 SA-AKI patients from center 1 were included in this study, comprising 464 males and 305 females. Among them, 101 patients (13.1%) required invasive ventilation. The sample size was optimal based on the enrolled variables and the number of cases requiring invasive ventilation. The training group consisted of 539 patients, while the internal validation group included 230 patients. There were no statistically significant differences in baseline characteristics between the two groups (Supplementary Table 1).

### Univariate analyses in the training group

Univariate analysis in the training group identified significant variables associated with invasive ventilation risk, including lactate levels, respiratory rate, peripheral oxygen saturation, D-dimer, prothrombin time, pro-BNP, albumin, and pulmonary infection (*P* < 0.001) (Table 1). Variance inflation factors (VIFs) were 1.352, 1.197, 1.130, 1.101, 1.078, 1.062, 1.054, and 1.057, respectively, indicating no multicollinearity. The Box-Tidwell test results yielded *P*-values of 0.767, 0.149, 0.146, 0.227, 0.956, 0.914, and 0.605 for lactate, pro-BNP, prothrombin time, D-dimer, albumin, peripheral oxygen saturation, and respiratory rate, respectively (*P* > 0.05), confirming a linear relationship with logit(p). These variables were therefore included in the multivariate logistic regression analysis.

**TABLE 1:** Univariate analysis between patients with ventilation and no ventilation in training population^a^.

Variables	No ventilation group (*n* = 470)	Ventilation group (*n* = 69)	*p*
Gender			0.712
Male	278 (59)	43 (62)	
Female	192 (41)	26 (38)	
Age (years)	74 (62, 83)	72 (59, 82)	0.739
Laboratory index^b^			
HS-CRP (mg/L)	142 (79, 200)	155 (55, 200)	0.922
Alanine transaminase (U/L)	23 (13, 42)	34 (17, 60)	0.005
Triglyceride (mmol/L)	3 (2, 4)	3 (2, 3)	0.018
Total bilirubin (umol/L)	12 (7, 20)	13 (8, 24)	0.095
Creatinine (umol/L)	181 (145, 265)	211 (159, 295)	0.053
Lactic acid (mmol/L)	2 (1, 3)	5 (3, 7)	<0.001
Pro-BNP (pg/mL)	2177 (855,6416)	5649 (1803, 18999)	<0.001
Cholinesterase (U/L)	4215 ± 1573	4048 ± 1831	0.473
Prothrombin time (s)	15 (14, 17)	16 (15, 20)	<0.001
D-dimer (mg/L)	4 (2, 7)	8 (3, 13)	<0.001
Potassium (mmol/L)			0.948
3.5–5.5	295 (63)	42 (61)	
<3.5	147 (31)	23 (33)	
>5.5	28 (6)	4 (6)	
Sodium (mmol/L)			0.006
135–145	227 (48)	35 (51)	
<135	221 (47)	24 (35)	
>145	22 (5)	10 (14)	
Magnesium (mmol/L)			0.14
0.75–1.25	305 (65)	43 (62)	
<0.75	160 (34)	23 (33)	
>1.25	5 (1)	3 (4)	
Calcium (mmol/L)			0.488
2.25–2.75	24 (5)	5 (7)	
<2.25	445 (95)	64 (93)	
>2.25	1 (0)	0 (0)	
White blood cell (×10^9^/L)			0.027
4–10	131 (28)	24 (35)	
<4	27 (6)	9 (13)	
>10	312 (66)	36 (52)	
Hemoglobin (×10^9^/L)			0.11
110–160	229 (49)	38 (55)	
<110	221 (47)	25 (36)	
>160	20 (4)	6 (9)	
Platelet (×10^9^/L)			0.208
100–300	327 (70)	43 (62)	
<100	118 (25)	19 (28)	
>300	25 (5)	7 (10)	
Albumin (g/L)	29 ± 5	27 ± 4	<0.001
Globulin (g/L)	27 (24, 31)	25 (20, 28)	0.001
Vital sign^b^			
SPO_2_ (%)	97 (95, 98)	93 (85, 97)	<0.001
Temperature (°C)			0.342
36–37.5	241 (51)	29 (42)	
<36	27 (6)	4 (6)	
>37.5	202 (43)	36 (52)	
MAP (mmHg)			0.049
70–105	287 (61)	36 (52)	
<70	126 (27)	28 (41)	
>105	57 (12)	5 (7)	
Heart rate (times/min)	98 (86, 114)	112 (95, 125)	0.001
Breathe rate (times/min)	20 (19, 22)	22 (20, 28)	<0.001
GCS	15 (15, 15)	15 (15, 15)	0.002
Coexisting disease			
Diabetes			0.916
No	375 (80)	56 (81)	
Yes	95 (20)	13 (19)	
Hypertension			0.163
No	240 (51)	42 (61)	
Yes	230 (49)	27 (39)	
Cerebral infarction			0.546
No	449 (96)	65 (94)	
Yes	21 (4)	4 (6)	
Cancer			0.357
No	405 (86)	56 (81)	
Yes	65 (14)	13 (19)	
Chronic lung disease			1
No	452 (96)	67 (97)	
Yes	18 (4)	2 (3)	
Chronic heart disease			0.697
No	458 (97)	67 (97)	
Yes	12 (3)	2 (3)	
Chronic liver disease			0.494
No	451 (96)	68 (99)	
Yes	19 (4)	1 (1)	
Chronic kidney disease			0.77
No	427 (91)	64 (93)	
Yes	43 (9)	5 (7)	
Leukemia			1
No	464 (99)	69 (100)	
Yes	6 (1)	0 (0)	
Infection site			
Intracranial infection			1
No	468 (100)	69 (100)	
Yes	2 (0)	0 (0)	
Lung infection			<0.001
No	351 (75)	30 (43)	
Yes	119 (25)	39 (57)	
Biliary infection			0.802
No	438 (93)	64 (93)	
Yes	32 (7)	5 (7)	
Urinary infection			0.478
No	374 (80)	58 (84)	
Yes	96 (20)	11 (16)	
Gastrointestinal infection			0.379
No	434 (92)	61 (88)	
Yes	36 (8)	8 (12)	

Callout:

*^a^*Continuous variables are described as medians and interquartile ranges due to non-normal distribution. Categories variables are analyzed by χ2 test and continuous variables are analyzed by Wilcoxon rank sum test;

*^b^*first examination index following admission. HS-CRP, high sensitivity -C reactive protein; pro-BNP, pro-brain natriuretic peptide; SPO_2_, pules oxygen saturation; MAP, mean arterial pressure; GCS, Glasgow coma score.

### Multivariate logistic regression and stepwise regression analyses

Multivariate and stepwise regression analyses identified lactate, pro-BNP, albumin, peripheral oxygen saturation, and pulmonary infection as independent risk factors for invasive ventilation within 48 h of admission (*P* < 0.05). These variables were incorporated into the final logistic model (Table 2).

**TABLE 2:** Multivariate logistic regression analysis and stepwise regression analysis of involved variables in training group.

	Multivariable logistic regression		Stepwise regression	
**Variables**	**OR (95% CI)**	***P*-value**	**OR (95% CI)**	***P*-value**
Lactic acid (mol/L)	1.151 (1.055–1.235)	<0.001	1.157 (1.074–1.250)	<0.001
Pro-BNP^a^ (pg/ml)	1.000 (1.000–1.000)	0.001	1.000 (1.000–1.000)	<0.001
Prothrombin time (s)	1.010 (0.967–1.041)	0.513	NA	NA
D-dimer (mg/L) SPO_2_ (%)	1.044 (0.994–1.094) 0.947 (0.914–0.976)	0.078 0.001	1.044 (0.994–1.093) 0.948 (0.915–0.976)	0.078 0.001
Albumin (g/L)	0.922 (0.867–0.980)	0.010	0.920 (0.866–0.978)	0.007
Breath rate (times/min)	1.001 (0.975–1.035)	0.899	NA	NA
Lung infection	2.909 (1.624–5.241)	<0.001	2.862 (1.603–5.136)	<0.001

Callout:*^a^*The exact pro-BNP in the multivariable logistic regression was 1.0000422 (1.0000165–1.0000673); the exact value for pro-BNP in the stepwise regression was 1.0000424 (1.0000171–1.0000672); SPO_2_, pules oxygen saturation.

### Nomogram construction

**FIGURE 1:**
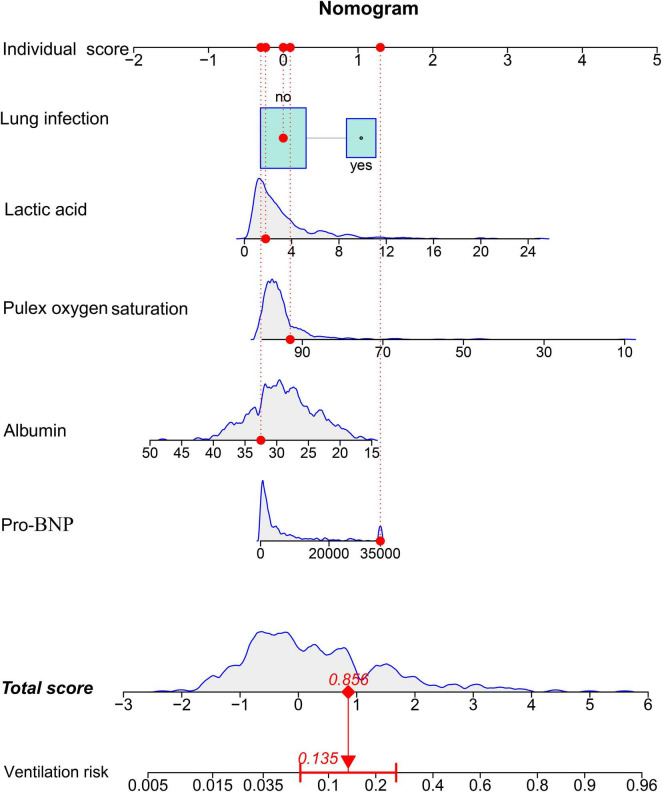
Risk-prediction nomogram for invasive ventilation within 48 h following admission in patients with sepsis.

A nomogram was developed based on the identified independent risk factors (Figure 1). Each factor got a score, with the total score used to estimate the probability of requiring invasive ventilation. In detail, a case was given in the figure as an example: the patient with pro-BNP of 35000 pg/mL, lactic acid of 1.8 mmol/L, presence of pulmonary infection, peripheral oxygen saturation of 93%, albumin of 32.5 g/L. A summed point was 0.856 and the predicted possibility of requiring ventilation was 0.135 using the lower total score lines.

### Model discrimination, calibration, and clinical effectiveness

**FIGURE 2:**
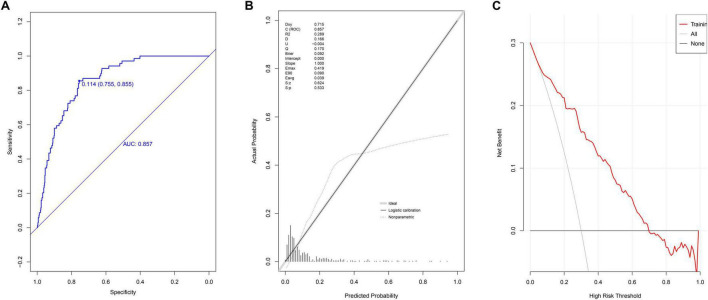
Evaluation of prediction model in modeling group. **(A)** ROC curves; **(B)** calibration chart; **(C)** DCA curves.

Receiver operating characteristic curve analysis yielded an AUC of 0.857 (95% CI: 0.818–0.897) (Figure 2A), demonstrating strong discriminative ability. The optimal cutoff value was 0.114. Model performance metrics included specificity of 75.5% (95% CI: 71.7%–79.4%), sensitivity of 85.5% (95% CI: 76.8%–92.8%), accuracy of 76.8% (95% CI: 76.7%–76.9%), PPV of 33.9% (95% CI: 26.9%–40.9%), and NPV of 97.3% (95% CI: 95.6%–98.9%). Calibration assessment using the Hosmer-Lemeshow test produced a *P*-value of 0.533, a Brier scaled score of 0.092, a calibration slope of 1.000, and an R^2^ value of 0.289 (Figure 2B). DCA curves showed the model consistently outperformed extreme threshold curves (Figure 2C), indicating high clinical utility.

### Internal model validation

**FIGURE 3:**
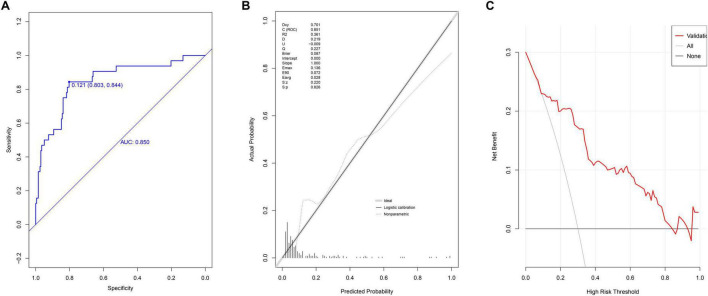
Evaluation of prediction model in internal validation group. **(A)** ROC curves; **(B)** calibration chart; **(C)** DCA curves.

In the validation group, the nomogram demonstrated strong discriminative ability, with an AUC of 0.850 (95% CI: 0.772–0.928) (Figure 3A). The optimal cutoff value was 0.121. The model’s performance metrics were as follows: specificity of 83.0% (95% CI: 74.8%–85.4%), sensitivity of 84.4% (95% CI: 71.9%–96.9%), accuracy of 80.9% (95% CI: 80.7%–81.0%), PPV of 40.9% (95% CI: 29.0%–52.8%), and NPV of 97.0% (95% CI: 94.3%–99.6%). Calibration analysis yielded a Hosmer-Lemeshow *P*-value of 0.826, a Brier scaled score of 0.087, a calibration slope of 1.000, and an R^2^ of 0.361 (Figure 3B). The DCA curve remained above the extreme curves (Figure 3C), confirming the model’s strong clinical utility.

### External model validation

**FIGURE 4:**
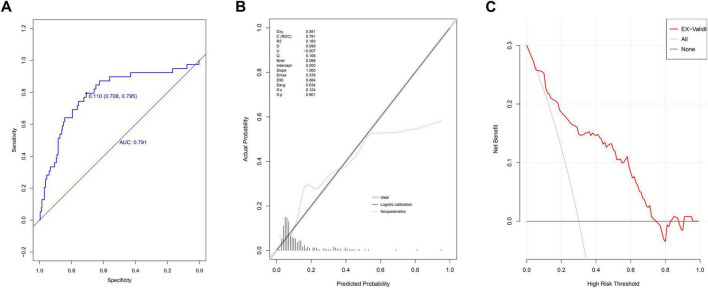
Evaluation of prediction model in external validation group. **(A)** ROC curves; **(B)** calibration chart; **(C)** DCA curves.

In the external validation group, the AUC of nomogram was 0.791 (95% CI: 0.710–0.872) (Figure 4A). At the cutoff value of 0.11, the model’s performance metrics were as follows: specificity of 73.0% (95% CI: 57.7%–88.0%), sensitivity of 82.1% (95% CI: 61.5%–94.8%), accuracy of 74.2% (95% CI: 61.8%–85.6%), PPV of 30.7% (95% CI: 23.3%–45.9%), and NPV of 96.5% (95% CI: 93.8%–98.9%). Calibration analysis yielded a Hosmer-Lemeshow *P*-value of 0.901, a Brier scaled score of 0.098, a calibration slope of 1.000, and an R^2^ of 0.183 (Figure 4B). The DCA curve remained above the extreme curves (Figure 4C).

### Comparison with the SOFA and NEWS scoring models

**FIGURE 5:**
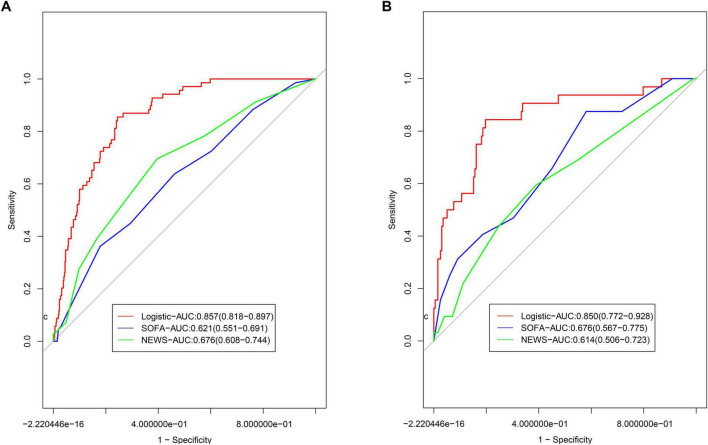
Comparison of ROCs for models. **(A)** Comparison to the models based on SOFA and NEWS scoring system in; **(B)** comparison to the models based on SOFA and NEWS scoring system in validation group.

In the training group, the AUC values for the SOFA and NEWS score models were 0.621 (95% CI: 0.551–0.691) and 0.676 (95% CI: 0.608–0.744), respectively (Figure 5A). Delong’s test revealed significant differences (Supplementary Table 2, *P* < 0.001), indicating superior discriminative ability of the nomogram. In the validation group, the AUC values for the SOFA and NEWS score models were 0.676 (95% CI: 0.567–0.775) and 0.614 (95% CI: 0.506–0.723), respectively (Figure 5B). Delong’s test results (*P* = 0.003 for SOFA and *P* < 0.001 for NEWS) further confirmed the nomogram’s superior predictive performance.

### Comparison with machine learning models

**FIGURE 6:**
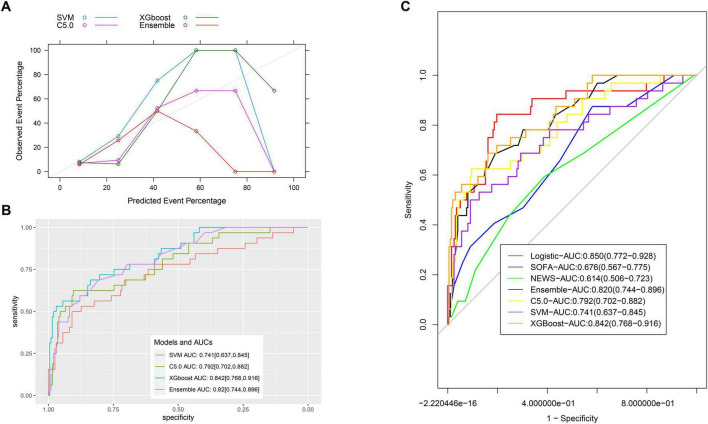
Comparison of ROCs for all models. **(A)** The calibration of machine learning models; **(B)** the AUC values for the C5.0, SVM, XGBoost, and ensemble models. **(C)** Comparison of ROCs for all models.

In the validation group, the calibration of machine learning models was less satisfactory (Figure 6A). The AUC values for the C5.0, SVM, XGBoost, and ensemble models were 0.792 (95% CI: 0.702–0.882), 0.741 (95% CI: 0.637–0.845), 0.842 (95% CI: 0.768–0.916), and 0.820 (95% CI: 0.744–0.896), respectively (Figure 6B). Comparisons with the nomogram using Delong’s test yielded *P*-values of 0.336, 0.1, 0.879, and 0.590, respectively, indicating no statistically significant differences (Figure 6C and Supplementary Table 2).

## Discussion

This retrospective study of SA-AKI patients identified lactate, pro-BNP, albumin, peripheral oxygen saturation, and pulmonary infection as independent risk factors for invasive ventilation within 48 h of admission. A predictive model based on these variables demonstrated strong performance.

Sepsis-associated acute kidney injury patients requiring invasive ventilation often experience prolonged hospital stays and increased mortality due to complications such as ventilator-induced lung injury and sedation-related pressure ulcers. The clinical utility of this predictive model lies in its ability to assess the short-term risk of invasive ventilation, enabling individualized interventions. These interventions include optimized fluid management using techniques such as Pulse Index Continuous Cardiac Output, pulmonary artery catheterization, or rigorous central venous pressure monitoring, as well as the timely application of non-invasive ventilation or high-flow oxygen therapy. Furthermore, the model enhances doctor-patient communication, particularly for high-risk individuals, reducing the risk of medical disputes.

Lactate, an indicator of oxygen metabolism, is produced by cells when oxygen consumption exceeds the supply and glycolysis occurs. Furthermore, lactate participates in an immune metabolic response during sepsis via serving as an anergy source of activated immune cells (20). In this way the high lactate levels found in this study stem from the metabolic reprogramming of sepsis, not only from Oxygen Delivery (DO2) Oxygen Consumption (VO2) imbalance (21), which was supported by the fact that mean arterial pressure was not enrolled in the final model. Similarly, the level of lactate in serum has been linked to poor outcomes and rapid progression to respiratory failure in sepsis cases (18). Pro-BNP, a marker of ventricular pressure, is synthesized and secreted by stretched cardiomyocyte. In patients with heart failure, ventricular wall extension, neurohormone activation and oxygen deficiency could result into increased secretion of BNP (22). In patients with sepsis, the BNP level increases due to myocardial injury, fluid overload, and renal impairment. Heart failure in sepsis patients causes lung edema and decreases the gas exchange function of lung, consequently leading to respiratory failure which need invasive ventilation (23, 24). This finding highlights the need for careful fluid management in SA-AKI patients with elevated pro-BNP levels. Albumin, a nutritional marker, reflects both respiratory muscle function and plasma oncotic pressure, with lower levels increasing the risk of pulmonary edema and respiratory failure (18, 25). Peripheral oxygen saturation directly correlates with oxygenation status, where lower levels indicate for a higher risk of intubation (26). Pulmonary infection increases the risk of secondary respiratory failure (27), which could be explained by impaired ventilation function of infected alveolar cell (28). Moreover, bacterial toxin and acidosis in infected cases result into pulmonary artery spasm and metabolic disorder, consequently causing injuries in multiple organs (such as respiratory failure) (29), Of course, an imbalance of inflammatory response in patients with sepsis would contribute to the occurrence of respiratory failure, which could be verified by including immune immunological molecules in the prediction model (e.g., TNF-alpha, IL-6 and procalcitonin) in the future. SaO_2_, PaO_2_ and PaO_2_/FiO_2_ ratio are essential for respiratory mechanics on spontaneous breathing, but not all these three indicators are routinely used in clinical practice, especially for patients who are not in intense care unit. Moreover, derived variables of PaCO2 are equally important as the ventilatory ratio (simply derived from ventilation per minute and PaCO2) is an important index to monitor respiratory failure and respiratory therapy (30). In this study, SPO2 is negatively associated with the risk of requiring mechanical ventilation, suggesting for the potential role of other related respiratory indicators in predicting invasive ventilation in patients with sepsis.

The nomogram outperformed the SOFA and NEWS score models in terms of discriminative ability, likely due to the inclusion of additional relevant clinical indicators. The SOFA and NEWS scores were developed earlier and do not incorporate certain variables now recognized as significant in clinical practice. Although some biomarkers, such as pro-BNP, may be costly, their clinical relevance justifies their inclusion. It is important to note that while SOFA and NEWS scores are valuable for assessing disease severity, they were not specifically designed to predict the need for mechanical ventilation.

Although machine learning models have advantages over than traditional models (31), they are comparable to our nomogram model for discriminative ability in this study. However, machine learning models suffer from the “black box” problem, limiting their interpretability. Given this limitation, logistic regression-based models are often preferred unless a significant performance advantage is demonstrated (32). Furthermore, less variables are enrolled in our model than that in the machine learning models, facilitating its usage in clinical practice. Finally, no priority on discrimination power of machine learning model over the logistic model could be explained by limited interactions between any two variables and small sample size for model comparison in this study (33, 34).

This study has several limitations. First, the retrospective design introduces potential selection bias caused by patients’ inclusion and data interpolated. Then, pulmonary infection was analyzed as a broad category without subclassification based on severity or etiology. Machine learning models were established at default parameters, and class imbalance might exist. Moreover, the information on whether patients received medical oxygen therapy or high flow oxygen therapy was not available in this study, although they could improve the prognosis of patients (35), However, was not available for enrolled cases in this study. Finally, some variables in the final model are not available for source limited regions, limited its broad usage in clinical practice.

## Conclusion

We developed a simple and effective predictive model incorporating key clinical parameters to estimate the risk of invasive ventilation in SA-AKI patients within 48 h of admission. The model demonstrated robust performance and could provide valuable insights to guide clinical decision-making, ultimately improving patients’ outcomes.

## Data Availability

The raw data supporting the conclusions of this article will be made available by the authors, without undue reservation.

## Supplementary material

Supplementary Figure 1 The flowchart of data processing in this study. **(A)** The flowchart of model establishment and validation; **(B)** the flowchart of variables selection before model establishment.
