# Supplementary material for: Development and validation of a predictive model for invasive ventilation risk within 48 hours of admission in patients with early sepsis-associated acute kidney injury
Source: Front Med (Lausanne). 2025 Jun 18;12:1577154. doi: 10.3389/fmed.2025.1577154 (PMC12213816; doi:10.3389/fmed.2025.1577154)
Supplement: Supplementary file 1 [file Table_1.docx]

**Supplementary 1. Baseline characteristics of the modeling population and validation population^a^**

| Variables | Training group  (n = 539) | Interval validation group (n = 230) | p |
| --- | --- | --- | --- |
| Gender |  |  | 0.549 |
| male | 321 (60) | 143 (62) |  |
| female | 218 (40) | 87 (38) |  |
| Age (years) | 73 (62, 83) | 73 (61, 82) | 0.617 |
| Laboratory index^b^ |  |  |  |
| HS-CRP (mg/L) | 143 (74, 200) | 149.01(83, 200) | 0.673 |
| Alanine transaminase  (U/L) | 24 (14, 45) | 23 (14, 40) | 0.449 |
| Triglyceride (mmol/L) | 3 (2, 4) | 3(2, 4) | 0.782 |
| Total bilirubin (umol/L) | 12 (7, 21) | 11 (8, 20) | 0.973 |
| Creatinine (umol/L) | 184 (148, 269) | 185(146, 278) | 0.777 |
| Lactic acid (mmol/L) | 2 (1, 4) | 2 (1, 4) | 0.242 |
| PRO-BNP (pg/ml) | 2315(919, 7373) | 2742(816, 9147) | 0.664 |
| Cholinesterase (U/L) | 4186(3052, 5175) | 3909(2786,5065) | 0.11 |
| Prothrombin time (s) | 15 (14, 17) | 16 (14, 17) | 0.375 |
| D-dimer (mg/L) | 4 (2, 8) | 4(2, 8) | 0.737 |
| Potassium (mmol/L) |  |  | 0.857 |
| 3.5-5.5 | 337 (63) | 141 (61) |  |
| <3.5 | 170 (32) | 73 (32) |  |
| >5.5 | 32 (6) | 16 (7) |  |
| Sodium (mmol/L) |  |  | 0.706 |
| 135-145 | 262 (49) | 105 (46) |  |
| <135 | 245 (45) | 109 (47) |  |
| >145 | 32 (6) | 16 (7) |  |
| Magnesium (mmol/L) |  |  | 0.445 |
| 0.75-1.25 | 348 (65) | 138 (60) |  |
| <0.75 | 183 (34) | 89 (39) |  |
| >1.25 | 8 (1) | 3 (1) |  |
| Calcium (mmol/L) |  |  | 0.175 |
| 2.25-2.75 | 29 (5) | 17 (7) |  |
| <2.25 | 509 (94) | 211 (92) |  |
| >2.25 | 1 (0) | 2 (1) |  |
| White blood cell (*10^9^/L) |  |  | 0.708 |
| 4-10 | 155 (29) | 73 (32) |  |
| <4 | 36 (7) | 15 (7) |  |
| >10 | 348 (65) | 142 (62) |  |
| Hemoglobin (*10^9^/L) |  |  | 0.061 |
| 110-160 | 267 (50) | 121 (53) |  |
| <110 | 246 (46) | 106 (46) |  |
| >160 | 26 (5) | 3 (1) |  |
| Platelet (*10^9^/L) |  |  | 0.48 |
| 100-300 | 370 (69) | 150 (65) |  |
| <100 | 137 (25) | 68 (30) |  |
| >300 | 32 (6) | 12 (5) |  |
| Albumin (g/L) | 29(26, 32) | 29(26, 32) | 0.179 |
| Globulin (g/L) | 27 (24, 30) | 27(23, 31) | 0.6 |
| Vital sign^b^ |  |  |  |
| SPO_2_ (%) | 96 (95, 98) | 97 (95, 99) | 0.052 |
| Temperature (℃) |  |  | 0.366 |
| 36-37.5 | 270 (50) | 123 (53) |  |
| <36 | 31 (6) | 17 (7) |  |
| >37.5 | 238 (44) | 90 (39) |  |
| MAP (mmHg) |  |  | 0.991 |
| 70-105 | 323 (60) | 139 (60) |  |
| <70 | 154 (29) | 65 (28) |  |
| >105 | 62 (12) | 26 (11) |  |
| Heart rate (times/min) | 100 (86, 116) | 98 (84, 116) | 0.294 |
| Breathe rate (times/min) | 20 (20, 22) | 20 (18, 22) | 0.062 |
| GCS | 15 (15, 15) | 15 (15, 15) | 0.245 |
| Coexisting disease |  |  |  |
| Diabetes |  |  | 0.268 |
| no | 431 (80) | 175 (76) |  |
| yes | 108 (20) | 55 (24) |  |
| Hypertension |  |  | 0.163 |
| no | 282 (52) | 107 (47) |  |
| yes | 257 (48) | 123 (53) |  |
| Cerebral infarction |  |  | 0.256 |
| no | 514 (95) | 214 (93) |  |
| yes | 25 (5) | 16 (7) |  |
| Cancer |  |  | 0.537 |
| no | 461 (86) | 192 (83) |  |
| yes | 78 (14) | 38 (17) |  |
| Chronic lung disease |  |  | 0.307 |
| no | 519 (96) | 217 (94) |  |
| yes | 20 (4) | 13 (6) |  |
| Chronic heart disease |  |  | 0.105 |
| no | 525 (97) | 218 (95) |  |
| yes | 14 (3) | 12 (5) |  |
| Chronic liver disease |  |  | 1 |
| no | 519 (96) | 221 (96) |  |
| yes | 20 (4) | 9 (4) |  |
| Chronic kidney disease |  |  | 1 |
| no | 491 (91) | 209 (91) |  |
| yes | 48 (9) | 21 (9) |  |
| Leukemia |  |  | 1 |
| no | 533 (99) | 228 (99) |  |
| yes | 6 (1) | 2 (1) |  |
| Infection site |  |  |  |
| Intracranial infection |  |  | 1 |
| no | 537 (100) | 230 (100) |  |
| yes | 2 (0) | 0 (0) |  |
| Lung infection |  |  | 0.931 |
| no | 381 (71) | 164 (71) |  |
| yes | 158 (29) | 66 (29) |  |
| Biliary infection |  |  | 0.987 |
| no | 502 (93) | 215 (93) |  |
| yes | 37 (7) | 15 (7) |  |
| Urinary infection |  |  | 0.786 |
| no | 432 (80) | 187 (81) |  |
| yes | 107 (20) | 43 (19) |  |
| Gastrointestinal infection |  |  | 0.525 |
| no | 495 (92) | 215 (93) |  |
| yes | 44 (8) | 15 (7) |  |
| NEWs score | 4 (3, 6) | 4 (3, 6) | 0.269 |
| SOFA score | 5 (4, 7) | 5 (3, 7) | 0.91 |

Callout: a, Continuous variables are described as medians and interquartile ranges due to non-normal distribution. Categories variables are analyzed by χ2 test and continuous variables are analyzed by Wilcoxon rank sum test; b, first examination index following admission.

HS-CRP, high sensitivity -C reactive protein; pro-BNP, pro-brain natriuretic peptide; SPO_2_, pules oxygen saturation; MAP, mean arterial pressure; GCS, Glasgow coma score.
