# Supplementary material for: Development and validation of a predictive model for invasive ventilation risk within 48 hours of admission in patients with early sepsis-associated acute kidney injury
Source: Front Med (Lausanne). 2025 Jun 18;12:1577154. doi: 10.3389/fmed.2025.1577154 (PMC12213816; doi:10.3389/fmed.2025.1577154)
Supplement: Supplementary file 2 [file Table_2.docx]

Supplementary 2. The comparison of model performances based on different method.

| Models | AUC | Accuracy | Sensitivity | Specificity | PPV | NPV | P (Delong test) |
| --- | --- | --- | --- | --- | --- | --- | --- |
| Nomogram | 0.850 (0.772-0.928) | 0.809 (0.807-0.810) | 0.844 (0.719-0.969) | 0.830 (0.748-0.854) | 0.409 (0.290-0.528) | 0.970 (0.943-0.996) | Reference |
| SOFA | 0.676 (0.567-0.775) | 0.488 (0.480-0.485) | 0.875 (0.760-0.990) | 0.419 (0.350-0.488) | 0.196 (0.131-0.261) | 0.954 (0.910-0.998) | 0.003 |
| NEWS | 0.614 (0.506-0.723) | 0.609 (0.607-0.611) | 0.594 (0.424-0.764) | 0.611 (0.543-0.679) | 0.198 (0.118-0.278) | 0.903 (0.853-0.953) | <0.001 |
| C5.0 | 0.792 (0.702-0.882) | 0.870 (0.591-0.913) | 0.625 (0.469-0.906) | 0.909 (0.535-0.965) | 0.528 (0.242-0.720) | 0.939 (0.915-0.974) | 0.336 |
| SVM | 0.741 (0.637-0.845) | 0.809 (0.591-0.891) | 0.656 (0.406-0.906) | 0.849 (0.561-0.944) | 0.375 (0.224-0.613) | 0.934 (0.904-0.974) | 0.1 |
| XGBoost | 0.842 (0.768-0.916) | 0.835 (0.522-0.939) | 0.719 (0.500-1.000) | 0.848 (0.444-0.990) | 0.442 (0.224-0.661) | 0.950 (0.923-1.000) | 0.879 |
| Ensemble | 0.820 (0.744-0.896) | 0.804 (0.552-0.900) | 0.719 (0.531-0.969) | 0.818 (0.490-0.949) | 0.391 (0.228-0.653) | 0.948 (0.920-0.987) | 0.590 |
