# Supplementary figures and images for: Development and validation of a predictive model for invasive ventilation risk within 48 hours of admission in patients with early sepsis-associated acute kidney injury
Source: Front Med (Lausanne). 2025 Jun 18;12:1577154. doi: 10.3389/fmed.2025.1577154 (PMC12213816; doi:10.3389/fmed.2025.1577154)

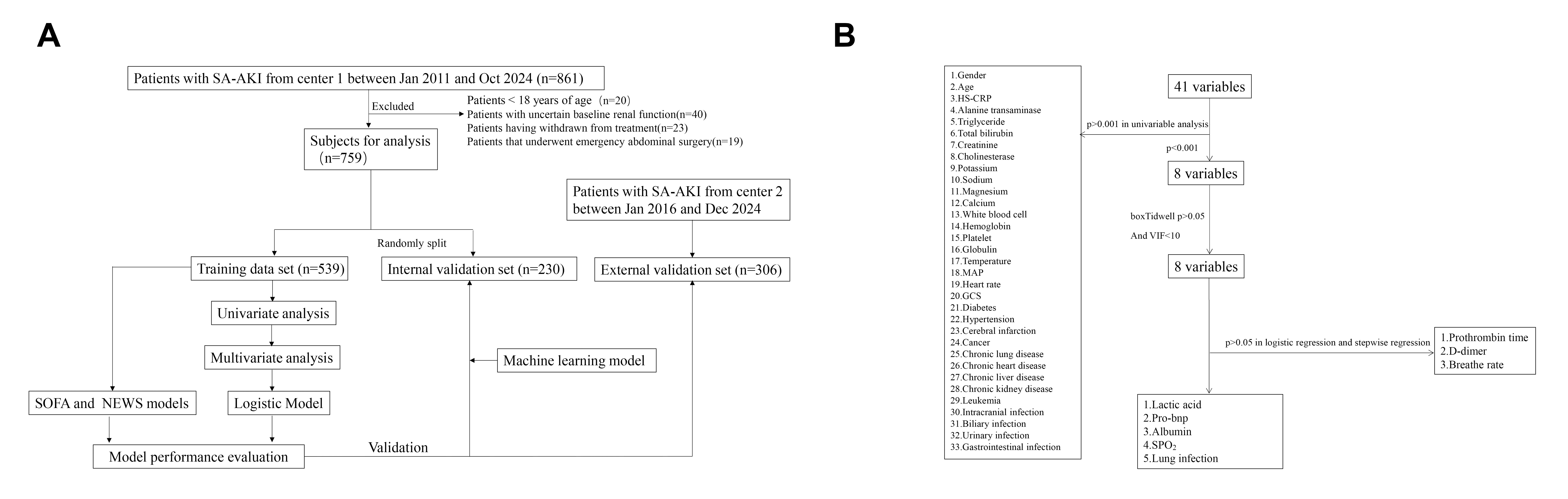

Supplement: Supplementary Figure 1 — The flowchart of data processing in this study. (A) The flowchart of model establishment and validation; (B) the flowchart of variables selection before model establishment. [file Image_1.tif]
